# Complete Genome Sequence of Mycobacterium avium subsp. *avium* Chester (DSM 44156)

**DOI:** 10.1128/MRA.01549-19

**Published:** 2020-02-13

**Authors:** Ralph Goethe, Kristin Laarmann, Cathrin Spröer, Boyke Bunk

**Affiliations:** a Institute for Microbiology, Department of Infectious Diseases, University of Veterinary Medicine Hannover, Hanover, Germany; b Leibniz Institute DSMZ-German Collection of Microorganisms and Cell Cultures, Braunschweig, Germany; c German Centre of Infection Research (DZIF), Partner Site Hannover-Braunschweig, Braunschweig, Germany; Broad Institute

## Abstract

Here, we announce the complete genome sequence of the Mycobacterium avium subsp. *avium* strain DSM 44156, also deposited as ATCC 25291 and TMC 724. The reference strain was originally described as a serotype 2 strain isolated from a hen by F. D. Chester in 1901.

## ANNOUNCEMENT

Mycobacterium avium is the nontuberculous mycobacterial species which is most frequently isolated from animals and humans (1). Due to phenotypical and especially molecular typing techniques, Mycobacterium avium was classified into four subspecies, M. avium subsp. *avium*, M. avium subsp. *paratuberculosis*, M. avium subsp. *silvaticum*, and M. avium subsp. *hominissuis* (2, 3). However, despite belonging to the same species, M. avium subspecies differ considerably, e.g., in growth behavior, genomic organization, pathogenicity, and host preference. M. avium subsp. *avium*, M. avium subsp. *silvaticum*, and M. avium subsp. *paratuberculosis* are obligate pathogens. M. avium subsp. *avium* causes avian tuberculosis, a fatal mycobacteriosis, in birds (4). M. avium subsp. *silvaticum* causes mycobacteriosis in pigeons (2, 5). M. avium subsp. *paratuberculosis* is the causative agent of paratuberculosis, a chronic, progressive, and fatal enteritis in ruminants (6). In contrast, M. avium subsp. *hominissuis* is considered an environmental bacterium and opportunistic pathogen for humans, pigs, and other species (1). Subspecies-specific large-sequence polymorphisms are suggested to be responsible for the individual features of each subspecies (7–9). Here, we provide the complete genome sequence of the M. avium subsp. *avium* reference strain DSM 44156, also deposited as ATCC 25291 and TMC 724, a serotype 2 strain originally isolated from liver of a diseased hen (10). The reference strain is one of the most intensively used strains for studying M. avium pathogenicity.

M. avium subsp. *avium* DSM 44156 was provided by the Leibniz Institute DSMZ and cultured in Middlebrook 7H9 medium with glycerol, oleic acid, albumin, and dextrose at 37°C to the late exponential growth phase. DNA was isolated using a Genomic-tip 100/G (Qiagen, Hilden, Germany). A SMRTbell template library for long-read sequencing was prepared according to the instructions from Pacific Biosciences and sequenced on the PacBio RS II system (Menlo Park, CA, USA), taking one 240-minute movie, resulting in 50,721 reads with a mean (filtered) read length of 10,177 bp. Libraries for sequencing on an Illumina platform were prepared by applying a Nextera XT DNA library preparation kit with modifications (11) and sequenced on the NextSeq 500 platform (Illumina, San Diego, CA, USA), leading to 8.8 million reads. Long-read genome assembly was performed with the RS_HGAP_Assembly.3 protocol included in SMRT Portal 2.3.0 by applying a target genome size of 10 Mbp, including quality filtering with a minimum subread length of 500 bp and a minimum polymerase read quality of 0.8. The chromosomal contig was circularized, and in particular, artificial redundancies at the ends of the contigs were removed and adjusted to *dnaA*. Identification of redundancies and the replication gene was done based on BLAST, and circularization and rotation to the replication genes were performed using the genomecirculator.jar tool (https://github.com/boykebunk/genomefinish). Error correction was performed by mapping the Illumina short reads onto the finished genome using the Burrows-Wheeler Aligner (BWA) 0.6.2 in paired-end (sample) mode using default settings (12) with subsequent variant and consensus calling using VarScan 2.3.6 (13). Genome annotation was based on Prokka 1.8 (14) with subsequent manual curation.

The complete circular chromosome comprises 4,956,929 bp with 4,626 predicted genes and 4,529 coding sequences (CDS), 301 of which carry signal peptide sequences. The GC content was determined to be 69.3%. The genome harbors 3 rRNA genes and 58 tRNA genes. The average (long-read) sequencing depth is 87×. Sequencing was conducted from authentic biological material grown from an ampule at DSMZ.

### Data availability.

The genome sequence has been deposited at NCBI GenBank under accession number CP046507. The version described in this paper is the first version, CP046507.1. The raw sequence reads have been submitted to the NCBI SRA under the accession number of the corresponding BioProject, PRJNA591110.

## References

[1] Rindi L, Garzelli C 2014 Genetic diversity and phylogeny of Mycobacterium avium. Infect Genet Evol 21:375–383. doi:10.1016/j.meegid.2013.12.007. 24345519

[2] Thorel MF, Krichevsky M, Lévy-Frébault VV 1990 Numerical taxonomy of mycobactin-dependent mycobacteria, emended description of Mycobacterium avium, and description of Mycobacterium avium subsp. avium subsp. nov., Mycobacterium avium subsp. paratuberculosis subsp. nov., and Mycobacterium avium subsp. silvaticum subsp. nov. Int J Syst Bacteriol 40:254–260. doi:10.1099/00207713-40-3-254. 2397193

[3] Mijs W, de Haas P, Rossau R, Van der Laan T, Rigouts L, Portaels F, van Soolingen D 2002 Molecular evidence to support a proposal to reserve the designation Mycobacterium avium subsp. avium for bird-type isolates and “M. avium subsp. hominissuis” for the human/porcine type of M. avium. Int J Syst Evol Microbiol 52:1505–1518. doi:10.1099/00207713-52-5-1505. 12361252

[4] Tell LA, Woods L, Cromie RL 2001 Mycobacteriosis in birds. Rev Sci Tech 20:180–203. doi:10.20506/rst.20.1.1273. 11288511

[5] Soltys MA, Wise DR 1967 Atypical mycobacterium in tuberculosis-like lesions in wood pigeons. J Pathol Bacteriol 93:351–352. doi:10.1002/path.1700930139. 6029773

[6] Harris NB, Barletta RG 2001 Mycobacterium avium subsp. paratuberculosis in veterinary medicine. Clin Microbiol Rev 14:489–512. doi:10.1128/CMR.14.3.489-512.2001. 11432810 PMC88986

[7] Alexander DC, Turenne CY, Behr MA 2009 Insertion and deletion events that define the pathogen Mycobacterium avium subsp. paratuberculosis. J Bacteriol 191:1018–1025. doi:10.1128/JB.01340-08. 19028885 PMC2632067

[8] Semret M, Turenne CY, de Haas P, Collins DM, Behr MA 2006 Differentiating host-associated variants of Mycobacterium avium by PCR for detection of large sequence polymorphisms. J Clin Microbiol 44:881–887. doi:10.1128/JCM.44.3.881-887.2006. 16517871 PMC1393138

[9] Turenne CY, Wallace R Jr, Behr MA 2007 Mycobacterium avium in the postgenomic era. Clin Microbiol Rev 20:205–229. doi:10.1128/CMR.00036-06. 17428883 PMC1865596

[10] Engbaek HC, Runyon EH, Karlson AG 1971 Mycobacterium avium Chester: designation of the neotype strain. Int J Syst Bacteriol 21:192–196. doi:10.1099/00207713-21-2-192.

[11] Baym M, Kryazhimskiy S, Lieberman TD, Chung H, Desai MM, Kishony R 2015 Inexpensive multiplexed library preparation for megabase-sized genomes. PLoS One 10:e0128036. doi:10.1371/journal.pone.0128036. 26000737 PMC4441430

[12] Li H, Durbin R 2009 Fast and accurate short read alignment with Burrows-Wheeler transform. Bioinformatics 25:1754–1760. doi:10.1093/bioinformatics/btp324. 19451168 PMC2705234

[13] Koboldt DC, Zhang Q, Larson DE, Shen D, McLellan MD, Lin L, Miller CA, Mardis ER, Ding L, Wilson RK 2012 VarScan 2: somatic mutation and copy number alteration discovery in cancer by exome sequencing. Genome Res 22:568–576. doi:10.1101/gr.129684.111. 22300766 PMC3290792

[14] Seemann T 2014 Prokka: rapid prokaryotic genome annotation. Bioinformatics 30:2068–2069. doi:10.1093/bioinformatics/btu153. 24642063

